# Amperometric Enzyme Sensor to Check the Total Antioxidant Capacity of Several Mixed Berries. Comparison with Two Other Spectrophotometric and Fluorimetric Methods

**DOI:** 10.3390/s150203435

**Published:** 2015-02-03

**Authors:** Mauro Tomassetti, Maruschka Serone, Riccardo Angeloni, Luigi Campanella, Elisa Mazzone

**Affiliations:** Department of Chemistry, “Sapienza” University of Rome, p.le A. Moro 5, 00185 Rome, Italy; E-Mails: m.serone@live.it (M.S.); luigi.campanella@uniroma1.it (L.C.); mazzone.elisa@libero.it (E.M.)

**Keywords:** antioxidant capacity, amperometric measurement, biosensor, fluorimetric, spectrophotometric, methods

## Abstract

The aim of this research was to test the correctness of response of a superoxide dismutase amperometric biosensor used for the purpose of measuring and ranking the total antioxidant capacity of several systematically analysed mixed berries. Several methods are described in the literature for determining antioxidant capacity, each culminating in the construction of an antioxidant capacity scale and each using its own unit of measurement. It was therefore endeavoured to correlate and compare the results obtained using the present amperometric biosensor method with those resulting from two other different methods for determining the total antioxidant capacity selected from among those more frequently cited in the literature. The purpose was to establish a methodological approach consisting in the simultaneous application of different methods that it would be possible to use to obtain an accurate estimation of the total antioxidant capacity of different mixed berries and the food products containing them. Testing was therefore extended to also cover jams, yoghurts and juices containing mixed berries.

## 1. Introduction

The antioxidant activity of different plant food species is increasingly used to combat oxidative stress in the human body caused by the accumulation of reactive chemical species [1], in particular those of oxygen, the so called Reactive Oxygen Species (ROS) [2]. Oxidative stress is known to be caused by several different factors, including radiation, pollution, cigarette smoking, alcohol, some drugs, overexposure to sun, and how all this consequently leads to an excessive production of free radicals [3] including in particular the superoxide radical which also acts as an intermediate product in many radical reactions.

Free radicals often trigger chain reactions that are harmful to the body, giving rise to serious cardiovascular disorders (arteriosclerosis, hypertension, heart failure), but can also cause degenerative nervous disorders (Alzheimer's, Parkinson's) and early ageing and tumors [1].

When taken in suitable concentrations, antioxidants can prevent or inhibit the oxidation of a target biomolecule. Dieticians actually prescribe diets containing antioxidants for conditions resistant to conventional pharmacological treatments [1].

Two different kinds of antioxidant have been discovered so far—endogenous and exogenous antioxidants. Endogenous antioxidants, that is, those produced by the body, mainly comprise enzymatic complexes, such as catalase, glutathione peroxidase, superoxide dismutase (SOD), but also other molecules such as uric acid and lipoic acid [4].

Exogenous antioxidants, that is, those that have to be administered in the diet, consist of several vitamins (A, C, E), carotenoids, flavonoids and polyphenols [5–8]. Moreover, in order to achieve their task, antioxidants must be present in relatively low concentrations. Indeed at higher concentrations they may turn into pro-oxidants and actually facilitate radical formation [2]. Keeping a close watch on the amount of antioxidants consumed and their action against free radicals is therefore of the essence in maintaining a condition of health. Numerous epidemiological studies have revealed the existence of a positive correlation between eating fruit and the reduction of heart disorders, cancer and other degenerative pathologies [9]. The various kinds of fruit investigated and described in the literature notably include mixed berries, which have a strong antioxidant capacity [10]. These grow on plants, that develop spontaneously in woods and forests, have high nutritive values; they are rich in vitamins, carotenoids, polyphenols, anthocyanins, ascorbic acid and also in mineral salts, such as potassium and magnesium [11].

In view of their ready availability, low cost and the wide use made of them due to the antioxidants they are known to contain, the focus of the present research is the study of the total antioxidant capacity of several of the more common mixed berries and the food products containing them. To do this they were analysed using an electroenzymatic method, in practice involving a superoxide dismutase (SOD) amperometric biosensor recently developed in our laboratory [12–14]. The results were then compared with those obtained using the well-known spectrophotometric method [15] with *N,N*-dimethyl-p-phenylenediamine (DMPD-FeCl_3_) and also with the “Oxygen Radical Absorbance Capacity” (ORAC) spectrofluometric method [16,17], which will be described below and are widely used to determine the total antioxidant capacity, for instance in various different kinds of vegetable samples [14].

## 2. Experimental Section

### 2.1. Samples

The mixed berries analysed and some food products obtained from them are listed in Table 1. All the test samples and their derivatives were purchased in local shops in Rome (Italy). All three jams analysed also contained sugar, fruit pectin and lemon juice. The yoghurt containing mixed berries also contained sugar, blackberry juice 3%, strawberry juice 3%, raspberry juice 2%, blueberry juice 0.5%, thickeners E414, E1422, pectin, aromas, colouring E120, acidity correctors E331 and E330. The yoghurt produced by a large Italian food manufacturer also contained saccharose, which is actually absent in natural fruit. Blueberry juice also contained citric acid and ascorbic acid, which are also known to have strong antioxidant properties.

### 2.2. Reagents and Chemicals

Xanthine oxidase 0.39 U·mg^−1^ and cellulose acetate were supplied by Fluka AG (Buchs, Switzerland). 2,2′-Azobis(2-amidinopropane) dihydrochloride (AAPH) was supplied by Waco Chem (Richmond, VA, USA). Potassium dihydrogenphosphate, sodium acetate and sodium hydrogenphosphate were supplied by Carlo Erba (Milan, Italy). Polyvinylacetate, acid-2-carboxy-6-hydroxy-2,5,7,8-tetramethylchroman (Trolox) was supplied by Aldrich (Steinheim, Germany). Xanthine (2,6-dihydroxypurine) sodium salt, *N,N*-dimethyl-*p*-phenylenediamine, ethylenediamine tetraacetic acid (EDTA) sodium salt, superoxide dismutase 4980 U·mg^−1^, ferric chloride, dialysis membrane (art. D-9777), and β-phycoerythrin were supplied by Sigma (Milan, Italy).

### 2.3. Apparatus

The following were used in the present research: an amperometric electrode mod. 4000-1 from Universal Sensor Inc. (New Orleans, LA, USA), coupled to an Amel potentiostat mod. 551, connected to an Amel differential electrometer, mod. 631 and to an Amel analog recorder, mod. 868 (all from Amel, Milan, Italy); Ultra-Turrax homogenizer mod. T8 by Ika Labortechnik; mill, A 10 Yellow line (IKA Works Inc., Staufen, Germany).

UV-VIS Lambda 5 spectrophotometer (Perkin Elmer, Waltham, MA, USA), provided with a printer; mod. GLP 22 pH meter (Crison, Barcelona, Spain); lastly a Perkin–Elmer spectrofluorimeter, mod. LS-5, equipped with a Perkin–Elmer recorder, mod. 561.

### 2.4. Methods

#### 2.4.1. Amperometric Method Based on a Superoxide Dismutase (SOD) Biosensor

##### Assembly of SOD Biosensor

**Figure 1.**
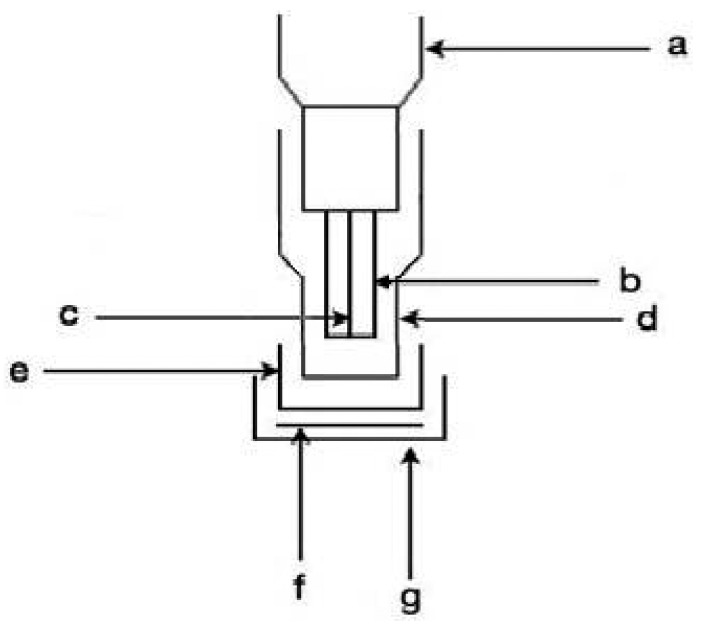
Assembly diagram of the SOD biosensor. **a**: electrode body; **b**: Ag/AgCl/Cl^−^ cathode; **c**: platinum anode (+ 650 mV); **d**: plastic cap; **e**: cellulose acetate membrane; **f**: Kappa-carrageenan disk containing SOD enzyme; **g**: dialysis membrane.

The biosensor used for superoxide radical determination (Figure 1) was constructed by coupling a transducer (an amperometric electrode for hydrogen peroxide) with the enzyme superoxide dismutase (SOD) (from bovine liver) immobilized in Kappa-carrageenan gel. The gel containing the enzyme was sandwiched between two membranes. The internal one, of cellulose acetate, was very thin (0.2 mm thick), while the external one consisted of a commercial dialysis membrane (A-9777). The entire assembly was fixed to the electrode by means of an O-ring. The electrode consisted of a platinum anode (to which a constant potential of + 650 mV was applied) and an Ag/AgCl/Cl^−^ cathode.

The outer dialysis membrane not only ensured the enzymatic membrane was securely fixed to the electrode but protected the enzyme against any bacterial attack.

##### Kappa-Carrageenan Gel Membrane

Kappa-carrageenan (0.2 g) was heated to 90 °C in distilled water (12 mL). The final solution (2% by weight) needed to be clear. The still hot gel was poured on to a Petri dish (diameter 4.5 cm), allowed to cool for 15 min in a refrigerator, and cut up into disks about 0.5 cm in diameter, which were then stored in a dessicator for at least 24 h. The disks thus obtained could be conserved in a refrigerator at +5 °C for about two months.

##### Enzyme Immobilization in the Kappa-Carrageenan Gel Disk

The enzymatic solution was prepared by dissolving 4 mg of SOD enzyme in 100 μL of buffer solution (sodium phosphate 0.05 M at pH 7.5); 25 μL of this solution were then taken and placed on a gel disk which had been previously placed in an Eppendorf test tube. The test tube was sealed and placed in a refrigerator at 4 °C until the enzymatic solution was completely absorbed by the gel disk.

##### Functioning of the Enzymatic-Amperometric Method

The superoxide radical (O_2_•^−^) was produced through the oxidation in aqueous solution of xanthine (C_5_H_4_N_4_O_2_) to uric acid (C_5_H_4_N_4_O_3_) in the presence of the enzyme xanthine oxidase:

(1) $$\underset{\left(\text{xanthine}\right)}{C_{5}H_{4}N_{4}O_{2}}+H_{2}O+O_{2}\overset{\text{xanthine oxidase}}{\to }\underset{\left(\text{uric acid}\right)}{C_{5}H_{4}N_{4}O_{3}}+2H^{+}+O_{2}^{•-}$$

The dismutation reaction of the superoxide radical, catalysed by the superoxide dismutase (SOD) immobilized on the electrode, released oxygen and hydrogen peroxide:

(2) $$O_{2}^{•-}+O_{2}^{•-}+2H^{+}\overset{\text{SOD}}{\to }H_{2}O_{2}+O_{2}$$

The released hydrogen peroxide was monitored by the amperometric sensor for H_2_O_2_. It was oxidized at the anode giving an amperometric signal (in nA) proportional to the concentration of the superoxide radical present in the solution.

The addition of a sample having antioxidant properties caused the signal to vary as the antioxidant species reacted with the superoxide radical and diminished the concentration of the latter in the solution, thus diminishing both the H_2_O_2_ produced and the intensity of the amperometric signal [12].

##### Measurements Using the Amperometric Biosensor

The amperometric biosensor was placed in a glass cell, thermostatted at room temperature, containing phosphate buffer (0.05 mol·L^−1^, pH 7.5, 10.0 mL) and allowed to stabilize under constant gentle stirring. A fixed quantity of the enzyme xanthine oxidase (20 μL, 5.74 mmol·L^−1^) was added to the solution; successive additions (at least three, each of 500 μL) of the xanthine solution (0.01 mol·L^−1^) were made, allowing the signal to stabilize between two successive additions before proceeding to read off the current variation caused by adding the xanthine solution and thus the hydrogen peroxide released.

Plotting the current variations *vs.* growing xanthine concentrations produced a straight calibration line which was used to calculate the slope (blank curve).

Exactly the same procedure was then followed, although also adding to the cell containing the phosphate buffer 1.0 mL of the sample to be tested. If the sample exhibited antioxidant properties, the observed amperometric signal was proportional to the reduction in superoxide radical concentration due to the reaction between the latter and the sample with antioxidant properties. The slope of the new straight line obtained was thus found to be less than that obtained in the absence of antioxidant. In other words the new calibration curve had a lower slope value than that of the blank curve. A comparison of the two slope values allowed the total antioxidant capacity of the test sample to be determined.

The antioxidant capacity was expressed in RAC (Relative Antioxidant Capacity) units, by means of the following expression:

(3) $$\text{Antiox capacity}=\left[1-({\text{m}}_{\text{b}}/{\text{m}}_{\text{a}})\right]\times 100$$

where m_a_ = slope of the straight line obtained by means of successive additions of xanthine (blank straight line). m_b_ = slope of the straight line obtained by means of successive additions of xanthine but in the presence of a sample with antioxidant properties.

##### Preparation of the Phosphate Buffer Solution

In order to prepare 250 mL of 0.05 mol·L^−1^ pH 7.5 phosphate buffer solution used to perform the measures, which contained also potassium chloride 0.01 mol·L^−1^ and EDTA 0.5 mmol·L^−1^, K_2_HPO_4_ (2.177 g), of KCl (0.186 g) and EDTA disodium salt (0.046 g), were weighed out. The solution was made up to 250 mL with distilled water and the pH checked, if necessary adjusting it with an HCl solution.

##### Preparation of the Xanthine Solution

Xanthine sodium salt (10 mg) were dissolved in distilled water (10 mL). If the resulting 5.74 mmol·L^−1^ solution of xanthine appeared cloudy, it had to be shaken until it became clear.

##### Sample Pretreatment

The samples with antioxidant properties were analysed as follows: sample (fruit or derived product, 1 g) was homogenized in phosphate buffer at pH 7.5 (6 mL) using a vortexer until a perfectly homogeneous solution was obtained. This solution was then diluted 1:10 with phosphate buffer, returned to the vortexer and again homogenized. This solution (1 mL) was taken and added to the measuring cell which already contained 10 mL of phosphate buffer.

#### 2.4.2. DMPD + FeCl_3_ Spectrophotometric Method

##### Principle of the Method

The cation radical obtained from the *N,N*-dimethyl-*p*-phenylenediamine dihydrochloride (DMPD), in the presence of a suitable oxidizing solution of Fe^3+^, at λ = 514 nm displays a peak absorption. The decrease in absorbance at this wavelength, recorded in the presence of the antioxidant sample, is a function of the latter's antioxidant capacity. Total antioxidant capacity is obtained by comparing the decrease in absorbance, due to the test sample, with that due to the 2-carboxy-6-hydroxy-2,5,7,8-tetramethylchroman acid (Trolox) taken as standard [15].

The principle on which the test is based is as follows: at acid pH and in the presence of a suitable oxidizing solution, the DMPD forms a stable coloured cation radical, according to the reaction:

(4) $${\left(CH_{3}\right)}_{2}NC_{6}H_{4}NH_{2_{\left(\text{colourless}\right)}}+Fe^{3+}\to {\left(CH_{3}\right)}_{2}NC_{6}H_{4}NH_{2_{\left(\text{purple}\right)}}^{•+}+Fe^{2+}$$

The antioxidant compounds (AOH), which are capable of transferring a hydrogen atom to the DMPD•^+^, produce a decolouring of the solution in proportion to their concentration, according to the following reaction:

(5) $${\left(CH_{3}\right)}_{2}NC_{6}H_{4}NH_{2_{\left(\text{purple}\right)}}^{•+}+AOH\to {\left(CH_{3}\right)}_{2}NC_{6}H_{4}NH_{3_{\left(\text{colourless}\right)}}^{+}+AO^{•}$$

The reaction is fast (taking less than 10 min) and the final absorbance, read off at λ = 514 mm, can be taken as a measure of the sample's total antioxidant capacity. The absorption spectrum of the cation radical solution displays two absorption peaks, one at 552 nm, and the other at 514 nm. The choice of wavelength (λ = 514 nm) is based on the fact that, at this wavelength, the method is found to be more sensitive.

##### Measurements

To a vessel containing acetate buffer (0.1 mol·L^−1^, pH 5.25, 100 mL) a solution of DMPD (0.1 mol·L^−1^, 1.0 mL) was added, together with a solution of FeCl_3_ (0.05 mmol·L^−1^, 0.2 mL) thus obtaining the purple-coloured cation radical DMPD•^+^. This solution (3 mL) was placed in a quartz cuvette with a 1.0 cm optical path. The absorbance was read off at λ = 514 nm. To this solution were then added sample (150 μL), or a 1.0 mg·mL^−1^ Trolox solution. This mixture was then constantly stirred at a temperature of 25 °C for 10 min, after which the absorbance was again read off at 514 nm. The reference cuvette contained only acetate buffer.

##### Data Processing

The results were expressed as percentage signal reduction, I_514_ (%), according to the expression:

(6) $${\text{I}}_{514}(\%)=\left(1-{\text{A}}_{f}/{\text{A}}_{0}\right)\times 100$$

where A_0_ = absorbance of the cation radical, recorded before adding the sample. A_f_ = absorbance recorded 10 min after addition of sample with antioxidant properties (whether test sample or standard Trolox solution). The antioxidant capacity of the samples tested was expressed in Trolox equivalent antioxidant capacity (TEAC) units in agreement with the method of Miller *et al.* [18], using the calibration straight line obtained using increasing quantities of Trolox, in view of the fact that the decrease in absorbance at λ = 514 nm was found to be linear between 0.2 and 11 μg of Trolox [13,15].

##### Solutions of Acetate Buffer, DMPD and FeCl_3_

To prepare 250 mL of 0.1 mol·L^−1^ pH 5.25 acetate buffer, glacial acetic acid (CH_3_COOH, 1.43 mL) was taken and potassium acetate (CH_3_COOK, 2.45 g) or sodium acetate (CH_3_COONa, 2.05 g) was weighed out. The solution was made up 250 mL with distilled water and the pH checked, adjusting it with HCl or NaOH if necessary.

To obtain the DMPD solution, *N,N*-dimethyl-*p*-phenylenediamine dihydrochloride (DMPD, 0.681 g) was dissolved in distilled water (50 mL); the solution had a DMPD concentration of 0.1 mol·L^−1^.

The FeCl_3_ solution was obtained by dissolving ferric chloride (0.0811 g) in distilled water (10 mL); the resulting solution had an FeCl_3_ concentration of 0.05 mol·L^−1^.

##### Sample Pretreatment

The samples with antioxidant properties were analysed as follows: fruit (1 g) was homogenized in phosphate buffer (pH 7.5, 6 mL) with the help of a vortexer until a homogeneous solution was obtained, which was then diluted 1:100 with phosphate buffer, returned to the vortexer and homogenized again. Then this solution (150 μL) was taken and added to the measuring cell containing acetate solution (0.1 mol·L^−1^, pH 5.25, 3 mL).

#### 2.4.3. ORAC Spectrofluorimetric Method

##### Principle of the Method

The protein β-phycoerythrin (β-PE), in the presence of free radicals or oxidizing species, can lose more than 90% of its fluorescence within 30 min. The addition of antioxidant species that react with free radicals inhibits the fluorescence decrease in this protein. The inhibition of free radical action is a function of the antioxidant capacity of the test sample [16]. In our case, 2,2′-azobis-(2-amidinopropane) dihydrochloride (AAPH) was used to generate the radicals.

##### Measurements

The wavelengths selected were 540 nm for excitation and 565 nm for emission. The sample (40 μL) was initially placed in the cuvette, to which were added phosphate buffer (75 mmol·L^−1^, pH 7, 790 μL), as well as β-phycoerythrin (18.3 nmol·L^−1^ in phosphate buffer, 730 μL). The cuvette was placed in the spectrofluorimeter and 30 s allowed to pass before taking the initial fluorescence reading. Later a further 20 μL of phosphate buffer and 20 μL of AAPH (320 mmol·L^−1^ in phosphate buffer) were added to the solution in the cuvette. The solution was allowed to mix and the fluorescence read off after 30 s and thereafter every 2 min for a total time of 70 min. A similar procedure was followed using a 20 μmol·L^−1^ solution of Trolox instead of the sample. Of course also a “blank” curve was recorded, that is without the addition of either sample or Trolox.

##### Data Processing

The final results were expressed as an “ORAC value” (micromoles of Trolox equivalent per litre of sample:

(7) $$\text{ORAC Value}=20k\left({\text{S}}_{\text{sample}}-{\text{S}}_{\text{blank}}\right)/\left({\text{S}}_{\text{trolox}}-{\text{S}}_{\text{blank}}\right)$$

*k* = dilution factor for the sample. *S* = integral of the fluorescence curve of the sample, or of the Trolox, or of the blank.

##### Sample Pretreatment

The sample was pretreated in exactly the same way as described in the case of the application of the superoxide dismutase biosensor method.

## 3. Results and Discussion

### 3.1. Results of Measurements

**Figure 2.**
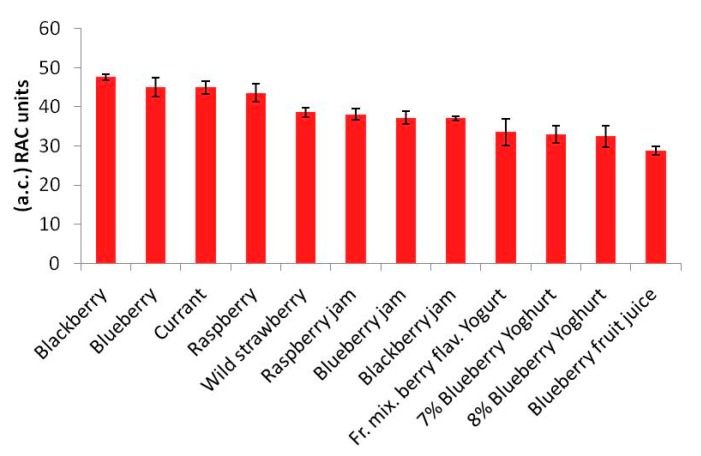
Histogram of antioxidant capacity values (a.c.), obtained using the biosensor method. Results expressed in RAC units.

Table 2 sets out the results obtained for all the samples tested using the amperometric-biosensor method and expressed in RAC units. The values in Table 2 are the means of three successive tests performed using the biosensor. The same results are shown as histograms in Figure 2.

The ranking of the different antioxidant capacities obtained for the various mixed berries and the food products containing them using the biosensor method, as clearly shown in Figure 2, was compared with the ranking obtained from the tests using the ORAC spectrofluorimetric method and the DMPD + FeCl_3_ spectrophotometric method.

**Figure 3.**
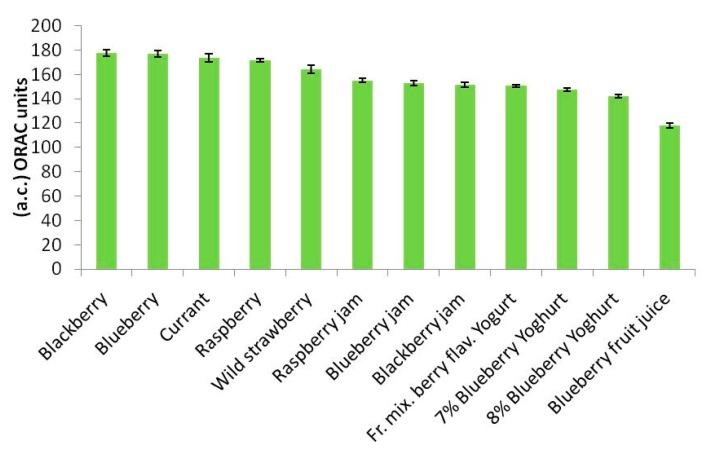
Histogram of antioxidant capacity values (a.c.), obtained using the spectrofluorimetric method. Results expressed in ORAC units.

The ORAC spectrofluorimetric method, despite recent criticism of it by the US Department of Agriculture [19], is the one best known and described in the literature, and in the past has been selected by the present [2,13,14] and other [20,21] authors as reference method as it usually produces reliable results (although it is very expensive for routine analysis). The tests were therefore performed also using this method and produced the results set out in Table 3, expressed in ORAC units. Figure 3 displays the results set out in Table 3 in the form of histograms.

**Figure 4.**
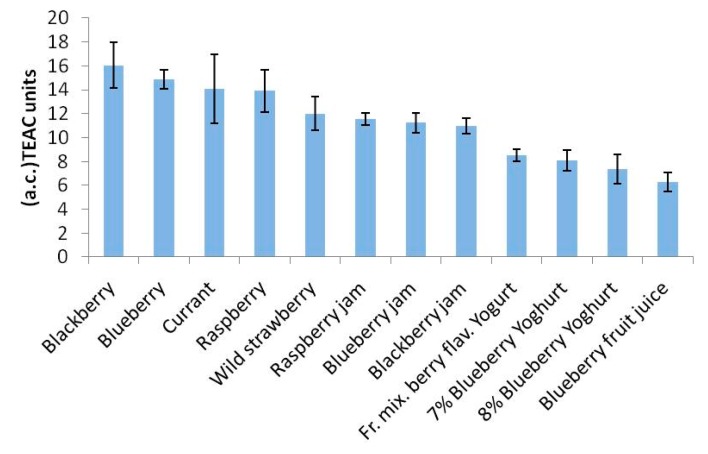
Histogram of antioxidant capacity values (a.c.), obtained using the spectrophotometric method. Results expressed in TEAC units.

Also in this case the results shown in Table 3 were performed by repeating each test in triplicate. Lastly, the results contained in Table 4, expressed in TEAC units, obtained using the spectrophotometric method, have also been presented as histograms in Figure 4.

**Figure 5.**
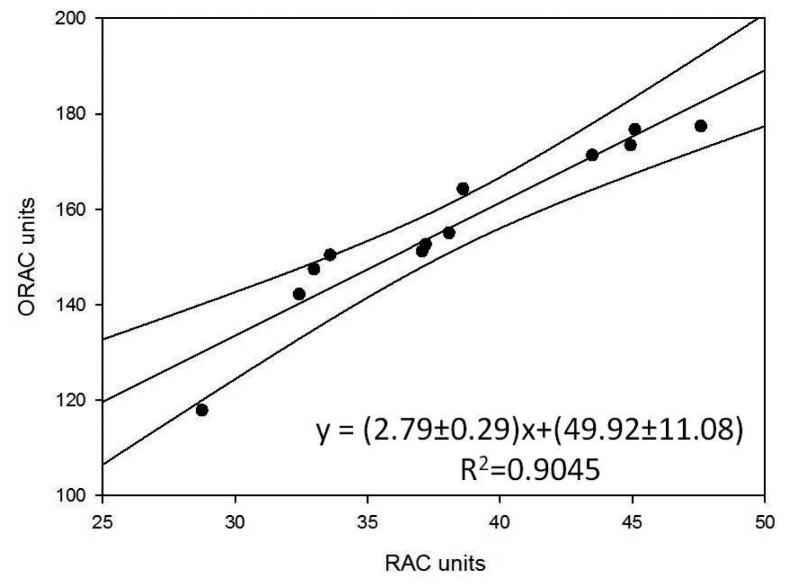
Correlation curve between the ORAC and SOD biosensor methods.

**Figure 6.**
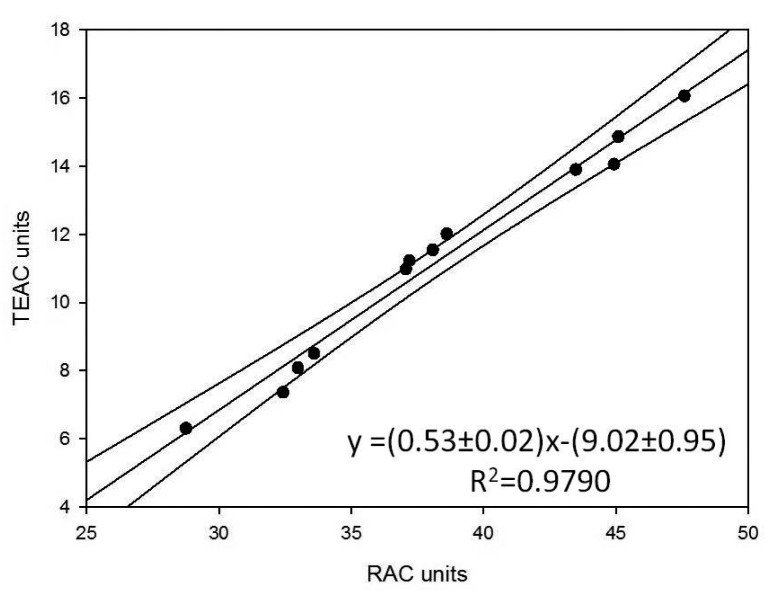
Correlation curve between the spectrophotometric and SOD biosensor methods.

**Figure 7.**
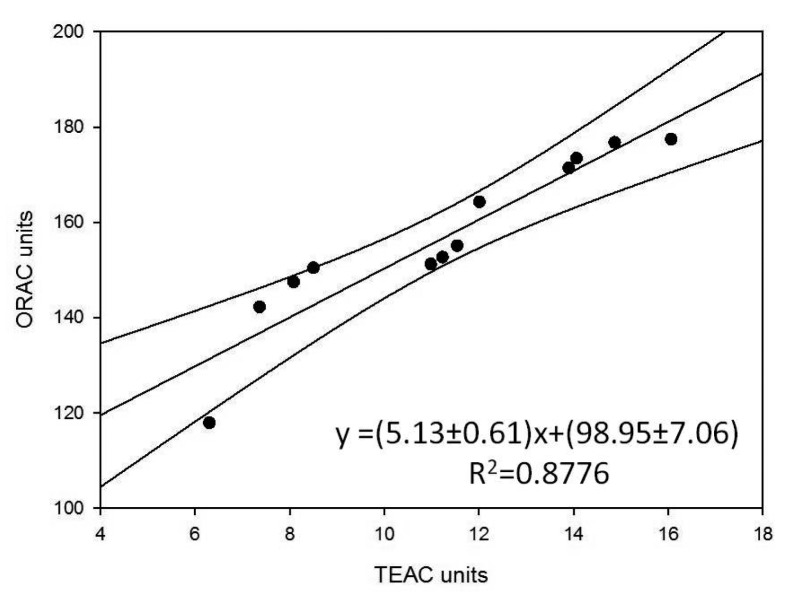
Correlation curve between the ORAC and spectrophotometric methods.

They also represent the mean of three successive applications of the same method. Comparison of the trends of the histograms in Figures 2, 3 and 4 shows how these trends are quite similar in all three cases. In order to analyse the correlation between the results using the three methods, the straight correlation lines referring to the ORAC and the SOD biosensor method, the spectrophotometric and the SOD biosensor method, and lastly the ORAC and the spectrophotometric method, respectively, are reported in Figures 5, 6 and 7.

This gives comparatively good correlations in the first two cases (*R*^2^ = 0.905 and 0.979) and a slightly less satisfactory, but acceptable correlation in the third case (*R*^2^ = 0.878). Furthermore, using the equations of the correlation lines thus obtained, it was possible to express in ORAC units all the results obtained using the other two methods (see Table 5) and apply the paired *t*-test, which showed in all three cases how |t_experimental_| < t_critical_ is always true (see t-experimental and t-critical values reported at the bottom of Table 5 for each of the three cases respectively). It follows that all the differences between the results found using the three methods are statistically non-significant. Lastly, for the purpose of comparing the precision of the three methods, the F-test actually shows that, except for a single case, F-experimental < F-critical is always true, so that the differences between the variance observed using three methods is not statistically significant. All this certainly confirms and supports the information found concerning the true antioxidant capacity of the analysed samples as well as the validity of the biosensor method developed by our research group.

### 3.2. Discussion

Since, as stated in the introduction, different species that have oxidizing properties exist, as well as different free radicals (O_2_•^−^, HO•, NO•, ROO•, *etc.*), and at the same time there are many antioxidant species (including enzymes such as SOD), it is also evident that each method for determining antioxidant capacity may have different characteristics and likely also different chemical reactivity.

It might consequently be deemed that each method can be considered valid only for a single radical or antioxidant species. This is partly why a number of different methods have therefore been developed, described in the literature [22–28], and considered suitable for determining the antioxidant capacity of different sample types. In reality, a good method should be able to measure the total antioxidant capacity [27] regardless of the type of radical produced. In addition a good method for determining the total antioxidant capacity should satisfy a number of requirements: as well as being applicable to any kind of sample, it would also have to be accurate and reproducible, comparatively simple and needing only instrumentation always available in any ordinary analytical laboratory. In the numerous literature reports, however, these needs, in particular the first one, are often found not to be adequately investigated.

In the present research, in order to clarify all this more thoroughly, it was preferred to apply simultaneously several different methods (possibly using different functioning modes to perform measurement in the same samples), in order to measure the total antioxidant capacity of complex matrices like those examined herein. For this reason, in the present research, three different methods were applied simultaneously: an electrochemical amperometric method, a spectrofluorimetric method and a spectrophotometric method.

The enzymatic-amperometric method developed by our research team [12–14,29] entails the use of an electrochemical sensor based on an amperometric electrode for H_2_O_2_ and on the enzyme superoxide dismutase. This method can be included among the methods for determining the total antioxidant capacity based on the superoxide radical and the superoxide dismutase enzyme [30].

The superoxide anion radical (O_2_•^−^) is one of the most common and important radicals produced in biological systems and in foodstuffs and behaves like the majority of radical species, which are extremely reactive and can be eliminated by enzymes such as superoxide dismutase or antioxidant agents such as ascorbic acid, polyphenol species and so on.

This analytical approach, based on SOD and the O_2_•^−^ radical is of great utility in the real-time determination of total antioxidant capacity and can be applied in different ways [30,31]. In the electroanalytical method described herein, the superoxide radical is produced in solution so that the superoxide dismutase enzyme, previously immobilized on the electrode surface, can catalyse the dismutation reaction of the superoxide anion radical, releasing molecular oxygen and hydrogen peroxide. The electro-oxidation of the hydrogen peroxide released generates the amperometric signal which is ultimately measured.

The use of a transducer for oxygen instead of one for hydrogen peroxide, although theoretically possible, was found not to be feasible in practice as the release of oxygen according to reaction (2), as set out in Section 2.4.1, would be offset by oxygen consumption, in accordance with reaction (1) as described in the same section.

The ORAC method represents a valid alternative to the Hydrogen Atom Transfer (HAT) method [20] used to measure the capacity of an antioxidant to inhibit free radicals deriving from hydrogen. Both methods can be included in the Total Radical Trapping Antioxidant Parameter (TRAP) group of methods. These methods are used to monitor the capacity of a compound to inhibit the reaction with peroxide radicals.

Lastly, the spectrophotometric method can be included in the Electron Transfer (ET) category [21]. This type of analytical method is based on the reactions produced by two compounds—an oxidizing agent and an antioxidant. The oxidizing species removes an electron from the antioxidant species, bringing about a characteristic change of colour in the solution; it is precisely this effect that is exploited to perform the test: the change in colour intensity is actually proportional to the concentration of the antioxidant species.

The most interesting findings made during the present research consisted of the fact that, as shown by the trends in the histograms set out in Figures 2, 3 and 4, the three methods, although relatively quite different, always display the same trend in their results for the different samples tested and so are in good reciprocal agreement: the correlation among them is actually more than acceptable, as shown in Figures 5, 6 and 7. This seems to show that, at least in the case of these three methods (although the same conclusion could probably be drawn also for other methods), they each actually do provide a measure of the total antioxidant capacity. In our view the main reason for this is that, as we have already postulated in previous research [32,33], in a complex real matrix all, or nearly all, the existing radical equilibria are closely linked. Consequently, the sudden increase or decrease of even one of these radicals is inevitably actually affected (at least to a certain degree) by the entire chain of radical reactions present, or that potentially exist, in the real matrix considered, and consequently also the content of other radicals varies and not just that involved in the specific test reaction. In our view, this is essentially the reason why even quite different analytical methods, provided they are always based on the measurement of the variation in radical species concentration, come up with relatively strongly correlated measures of total antioxidant capacity. The experimental results obtained in the present investigation, but also the similar ones obtained in previous investigations [2,13,14,29,34] actually appear to be in good agreement with this view.

Fresh mixed berries confirmed the expected antioxidant properties, in agreement with the abundant literature data. Blackberries were found to have a slightly greater antioxidant capacity for equal sample weight; this higher ranking was found in the results of all three different methods.

A good antioxidant capacity was found also in the commercial food products derived from the fresh fruits. Even though also ascorbic acid was present in several of these commercial products, the fact that, for equal weight of sample, fresh fruit and vegetables were found to have a higher antioxidant capacity than the commercial food products or food supplements containing them, always found in previous research [2,34,35], was confirmed also in the present work.

## 4. Conclusions

Despite the substantial differences among the three analytical methods used, the correspondence between the trends in the total antioxidant capacity values obtained using the three methods for the various mixed berries and food products containing them is without doubt a result that confirms and corroborates both the data obtained for the various test samples and the validity of the methods themselves, in particular of the amperometric-biosensor method, which as stated previously, was developed by our research team. The SOD amperometric biosensor method indeed confirmed its validity, displaying an excellent correlation with two other methods among the best known and most widely used by several researchers. The advantage of the SOD amperometric method over the ORAC method is essentially due to the fact that the latter is extremely expensive owing to the high cost of the β-phycoerythrin, while, as far as the spectrophotometric method is concerned, the advantage is essentially due to the fact that it is not subject to any of the chromatic interference that possibly occurs in real samples in which non-specific absorption at λ = 614 nm takes place.

## Figures and Tables

**Table 1.** Mixed berries and mixed berry food derivatives analysed in the present research.

**Berries**
Blackberry
Blueberry
Raspberry
Currant
Wild strawberry

**Prepared Foods**

Raspberry jam
Blackberry jam
Blueberry jam
Fresh Mixed Berry Flavoured Yogurt
(7%) Blueberry yogurt (from a dairy in the South Pontine area)
(8%) Blueberry yogurt (produced by a large Italian food manufacturer)
Blueberry fruit juice (produced by a large Italian food manufacturer)

**Table 2.** Repeated test results using the amperometric enzymatic method, values expressed in RAC units.

**Analyzed Product**	**Run I**	**Run II**	**Run III**	**Mean of the Three Measures**	**Standard Deviation**
Blackberry	47.92	46.76	48.07	47.58	0.72
Blueberry	45.63	42.44	47.19	45.09	2.4
Currant	46.90	43.85	44.02	44.92	1.7
Raspberry	41.52	45.90	43.02	43.48	2.3
Wild strawberry	38.20	39.91	37.71	38.61	1.2
Raspberry jam	36.98	39.72	37.56	38.08	1.4
Blueberry jam	38.71	35.46	37.39	37.19	1.6
Blackberry jam	37.02	37.60	36.58	37.06	0.51
Fresh Mixed Berry Flavoured Yogurt	37.01	33.50	30.26	33.59	3.4
(7%) Blueberry yogurt	35.85	32.22	30.86	32.98	2.2
(8%) Blueberry yogurt	35.56	31.69	30.02	32.42	2.8
Blueberry fruit juice	27.44	29.53	29.27	28.75	1.1

**Table 3.** Repeated test results using the ORAC spectrofluorimetric method,values expressed in ORAC units.

**Analyzed Product**	**Run I**	**Run II**	**Run III**	**Mean of the Three Measures**	**Standard Deviation**
Blackberry	178.00	177.52	176.78	177.43	2.5
Blueberry	177.67	177.40	175.23	176.77	2.7
Currant	174.61	174.08	171.60	173.43	3.2
Raspberry	172.19	170.97	170.99	171.38	1.4
Wild strawberry	167.72	163.98	161.14	164.28	3.3
Raspberry jam	152.98	156.83	155.30	155.04	1.9
Blueberry jam	151.27	155.16	151.54	152.66	2.2
Blackberry jam	149.98	153.50	150.13	151.20	2.0
Fresh Mixed Berry Flavoured Yogurt	151.34	150.12	149.78	150.41	0.82
(7%) Blueberry yogurt	146.49	149.01	146.90	147.47	1.4
(8%) Blueberry yogurt	141.97	143.56	141.04	142.19	1.3
Blueberry fruit juice	119.58	118.18	115.79	117.85	1.9

**Table 4.** Repeated test results using the DMPD-FeCl_3_ spectrophotometric method, values expressed in TEAC units.

**Analyzed Product**	**Run I**	**Run II**	**Run III**	**Mean of the Three Measures**	**Standard Deviation**
Blackberry	16.56	16.03	15.30	16.06	1.9
Blueberry	15.01	14.96	14.54	14.87	0.8
Currant	14.88	14.30	13.01	14.06	2.9
Raspberry	14.91	13.67	13.12	13.90	1.8
Wild strawberry	12.70	12.04	11.31	12.01	1.4
Raspberry jam	11.53	12.06	11.03	11.54	0.52
Blueberry jam	11.80	11.55	10.33	11.23	0.80
Blackberry jam	10.98	10.34	11.61	10.98	0.64
Fresh Mixed Berry Flavoured Yogurt	8.99	8.74	7.76	8.50	0.50
(7%) Blueberry yogurt	8.98	7.25	8.01	8.08	0.87
(8%) Blueberry yogurt	8.17	8.69	6.54	7.36	1.2
Blueberry fruit juice	6.59	5.39	6.91	6.30	0.80

**Table 5.** Statistical tests.

**Sample**	**Biosensor Method (ORAC Units) ± SD**	**Fluorimetric Method (ORAC units) ± SD**	**Spectrophotometric Method (ORAC Units) ± SD**	**(a) Biosensor Method *vs.* Fluorimetric Method**	**(B) Biosensor Method *vs.* Spectrophotometric Method**	**(c) Spectrophotometric Method *vs.* Fluorimetric Method**	**F-test: Two Tails Table, ν_1_ = ν_2_ = 2 (*p* = 95%)**	**Results of F-test N.S. = Not Significant S. = Significant**		

				**F_sp._**	**F_sp._**	**F_sp._**	**F_cr._**	**(a)**	**(b)**	**(c)**
Blackberry	182.50 ± 2.86	177.43 ± 6.25	181.42 ± 2.89	2.18	1.01	2.16	39	N.S.	N.S.	N.S.
Blueberry	175.56 ± 21.38	176.77 ± 7.29	175.31 ± 0.73	2.93	29.4	10.0	39	N.S.	N.S.	N.S.
Currant	175.08 ± 24.97	173.43 ± 10.24	171.15 ± 12.02	2.44	2.08	1.17	39	N.S.	N.S.	N.S.
Raspberry	171.07 ± 51.84	171.38 ± 1.96	170.33 ± 9.37	26.5	5.53	4.78	39	N.S.	N.S.	N.S.
Wild strawberry	157.50 ± 16.63	164.28 ± 10.89	160.62 ± 4.06	1.53	4.09	2.68	39	N.S.	N.S.	N.S.
Raspberry jam	156.03 ± 28.39	155.04 ± 3.61	158.21 ± 9.40	7.86	3.02	2.60	39	N.S.	N.S.	N.S.
Blueberry jam	153.55 ± 9.46	152.66 ± 4.84	156.62 ± 9.03	1.95	1.05	1.87	39	N.S.	N.S.	N.S.
Blackberry jam	153.18 ± 5.90	151.20 ± 4.00	155.34 ± 15.58	1.47	2.64	3.89	39	N.S.	N.S.	N.S.
Fresh Mixed Berry Flavoured Yogurt	143.51 ± 39.12	150.41 ± 0.67	142.60 ± 7.13	58.2	5.49	10.6	39	S.	N.S.	N.S.
7% Blueberry Yoghurt	141.82 ± 16.74	147.47 ± 1.96	140.44 ± 5.58	8.54	3.00	2.85	39	N.S.	N.S.	N.S.
8% Blueberry Yoghurt	140.25 ± 21.30	142.19 ± 1.69	136.75 ± 33.07	12.6	1.55	19.6	39	N.S.	N.S.	N.S.
Blueberry fruit juice	130.03 ± 49.28	117.85 ± 3.61	131.30 ± 13.21	13.7	3.73	3.66	39	N.S.	N.S.	N.S.

Paired t-test: two sided, ν = 11 (*p* = 95%)				t_sp._	t_sp._	t_sp._	t_cr._	Results of *t*-test		

				0.001	0.002	8.84 × 10^−5^	2.201	In all cases not significant (N.S.)		

F_sp._ = F_experimental_. F_cr._ = F_critical_. t_sp._ = t_experimental_. t_c_**_r_**_._ **=** t_critica_.
